# Muscle-strengthening exercise and sleep quality among a nationally representative sample of 23,635 German adults

**DOI:** 10.1016/j.pmedr.2020.101250

**Published:** 2020-11-25

**Authors:** Jason A. Bennie, Susanne Tittlbach

**Affiliations:** a Physically Active Lifestyles Research Group (USQ PALs), Institute for Resilient Regions, University of Southern Queensland, Springfield, Queensland, Australia; b Social and Health Sciences in Sport, Institute of Sport Science, University of Bayreuth, Bavaria, Germany

**Keywords:** Sleep health, Resistance training, Strength-promoting exercise, Epidemiology

## Abstract

Regular physical activity is a key modifiable non-pharmacological treatment to enhance sleep quality, a key predictor of optimal health and wellbeing. Most of the evidence on physical activity and sleep quality is based on studies assessing the effects of aerobic moderate-to-vigorous physical activity (e.g. brisk walking, cycling, jogging). Emerging clinical evidence suggests that muscle-strengthening exercise (e.g. push‐ups, using weight machines) may also be beneficial for sleep quality. However, since no studies have examined the associations between muscle-strengthening exercise with sleep quality among a large sample of community-dwelling adults, it is currently unknown how these findings translate to the population level. This study describes the associations between the weekly frequency of muscle-strengthening exercise and sleep quality among a nationally representative sample of German adults. Data were drawn for the 2014 German Health Update Survey (n = 23,635; ≥18 years). Validated survey items assessed muscle-strengthening exercise (times/week) and sleep quality (assessed via 4 point-scale: ‘*very poor*’ to ‘*good*’). Poisson regression with robust error variance was used to calculate prevalence ratios of (PR) across weekly muscle-strengthening exercise frequency (None [reference]; 1, 2, 3–4 and ≥ 5 times/week), adjusting for potential confounders (e.g. age, sex, socioeconomic status, self-rated health, smoking, alcohol, aerobic physical activity). Compared with those reporting none, any muscle-strengthening exercise was associated with a reduced prevalence of ‘*poor*’ (PR range: 0.77–0.83) and ‘*very poor*’ (PR range: 0.57–0.70) quality sleep. Future health behavior modification strategies to enhance sleep quality at the population-level should consider promoting muscle-strengthening exercise.

## Highlights

• 58.3% of the sample reported no muscle-strengthening exercise (MSE).
• Any MSE was associated with better sleep quality.
• Associations were strongest among those with the poorest sleep quality.
• Future population-level strategies to enhance sleep quality should promote MSE.

## 1 Introduction

Epidemiological evidence from prospective cohort studies links poor sleep quality to an increased risk of all-cause/disease-specific mortality and incident hypertension, cardiovascular diseases, diabetes and obesity (Itani et al., 2017, Jike et al., 2018). Physical activity is considered a key behavioral modification, non-pharmacological strategy to enhance sleep quality (U.S. Department of Health and Human Services, 2018). However, this evidence-base is principally generated from research assessing the benefits of regular aerobic moderate-to-vigorous physical activity/exercise (MVPA; e.g. walking, cycling, running) (U.S. Department of Health and Human Services, 2018), with limited research on other physical activity modalities.

While there is strong evidence from randomized controlled trials that aerobic MVPA has beneficial effects on multiple dimensions of sleep health, including total sleep time, sleep onset latency and sleep quality (U.S. Department of Health and Human Services, 2018), emerging clinical evidence suggests that muscle-strengthening exercise (e.g. using weight machines, push/sit-ups) may also be beneficial for enhancing sleep quality (Kovacevic et al., 2018). A 2018 systematic review of 13 small/short-duration randomized controlled trials, showed that muscle-strengthening exercise improved sleep quality, compared to no exercise (Kovacevic et al., 2018). However, since the available evidence on muscle-strengthening exercise and sleep quality is based on studies that contain small and non-representative samples, it is presently unknown how these findings translate to large population-based samples (Kovacevic et al., 2018). Research into the association between different physical activity modes and sleep quality among population samples is critical to inform future large-scale lifestyle modification interventions designed to improve sleep quality.

The purpose of this study is to describe the associations between weekly frequencies of muscle-strengthening exercise and sleep quality among a nationally representative sample of German adults.

## 2 Methods

### 2.1 Sample

Data were drawn from the 2014 German Health Update (hereafter: GEDA 2014). A detailed summary of the GEDA 2014 has been previously described (Lange et al., 2017). Conducted between November 2014 and July 2015, a two-stage stratified cluster sampling method was applied to recruit a nationally representative sample of adults aged ≥ 18 years. Initially, 90,102 invitations to participate were sent, with 24,016 responding (response rate = 27.6%) (Lange et al., 2017). In the present study, we excluded those who did not complete the sleep quality item (n = 381, 1.3% of the total sample).

### 2.2 Exposure - Muscle-strengthening exercise

To assess muscle-strengthening exercise, respondents were asked, “*In a typical week, on how many days do you carry out physical activities specifically designed to strengthen your muscles such as doing resistance training or strength exercises?*”. Respondents were requested to consider a selection of possible activities, such as strength exercises (using weights, elastic band, own body weight, etc.), knee bends (squats), push-ups (press-ups), sit-ups, etc. This item has shown evidence of ‘fair’ test–retest reliability (Intraclass Correlation Coefficient = 0.55) (Finger et al., 2015) and concurrent validity (using the ≥2 times/week threshold against all-cause mortality) (Stamatakis et al., 2017). Using categorisations consistent with previous studies (Bennie and Khan, 2018/10/24/ 2018., Bennie and Tittlbach, 2020/01/29/ 2020.), muscle-strengthening exercise was collapsed into: ‘0’; ‘1’; ‘2’; ‘3-4’ and ‘≥5 times/week’.

### 2.3 Outcome – sleep quality

To assess sleep quality, respondents were asked: “*Over the last 2 weeks, how often have you had trouble falling or staying asleep, or sleeping too much”.* Four response options were given: (i) ‘*Not at all*’ (recoded as ‘Good’); (ii) ‘*On some days*’ (recoded as ‘Fair’); (iii) ‘*More than half of the days*’ (recoded as ‘Poor’); and (iv) ‘*Almost every day*’(recoded as ‘Very poor’). This sleep quality indicator is from the Personal Health Questionnaire Depression Scale (PHQ-8) (Kroenke et al., 2009). The PHQ-8 has shown evidence of convergent validity (with the Health-Related Quality of Life questionnaire as the standard) (Kroenke et al., 2009), and hence is considered a reliable and valid tool to assess mental health/sleep quality in epidemiological studies.

### 2.4 Potential cofounders

Cofounders were selected a priori, with research showing that each is conceivably related to engagement in muscle-strengthening exercise (Bennie and Khan, 2018/10/24/ 2018., Bennie and Tittlbach, 2020/01/29/ 2020.) and sleep quality (Grandner, 2017). Sociodemographic (age, sex, socioeconomic position), lifestyle characteristics (aerobic MVPA, smoking, hazardous alcohol consumption [>20 g/day for women, >40 g/day for men], self-rated health, self-reported body mass index [BMI]) were assessed using validated survey items and scored using predetermined protocols (Lange et al., 2017). Socioeconomic position (low/medium/high) was assessed using the earlier validated, German-specific, Socioeconomic SES index (SES Index) (Lampert et al., 2013). In brief, the SES Index uses information from three constructs: [i] occupational status; [ii] formal education/vocational training; and [iii] equivalenced to net household income. As per protocol, a distribution-based distinction of three status groups is made for the analyses. With the low and high-status groups each encompassing 20% of the population, and the medium status group 60% (Lampert et al., 2013). Given that physical and mental health can affect sleep quality (Grandner, 2017), we also adjusted for those reporting “*being restricted by chronic disease in the past 6 months*” and by depressive symptom severity (assessed by PHQ-8) (Kroenke et al., 2009).

### 2.5 Statistical analysis

Analyses were conducted using the Complex Samples module of PASW Statistics 18. Weighting factors were included to correct for non-response to allow for valid population estimates (Eurostat European Commission, 2019). Descriptive statistics were used to describe the weighted percentages (%) across all potential cofounders, weekly frequency of muscle-strengthening exercise and sleep quality.

Generalized linear models with Poisson regression with robust error variance were used to calculate prevalence ratios (PR) assessing the associations between categories of sleep quality (dependant variable) across muscle-strengthening exercise (explanatory variable). The reference group was those reporting no muscle-strengthening exercise (0 times/week). Separate models were conducted for: (i) ‘*fair*’ (yes vs. no; reference [ref] = ‘*good*’); (ii) ‘*poor*’ (yes vs. no; ref = ‘*good*’); and (iii) ‘*very poor*’ (yes vs. no; ref = ‘*good*’). To account for the potential influence of sociodemographic/lifestyle characteristics and aerobic MVPA, we conducted three separate generalized linear models across each level of sleep quality: (i) ‘Model A’ (unadjusted); (ii) ‘Model B’ (adjusted for sex, age, socioeconomic position, hazardous alcohol consumption, smoking, self-rated health, BMI and being restricted by chronic disease in the past 6 months); and (iii) ‘Model C’ (adjusted for Model B + aerobic MVPA). Since muscle-strengthening exercise (Bennie and Khan, 2018/10/24/ 2018., Bennie and Tittlbach, 2020/01/29/ 2020.), and sleep quality differ by sex and age (Grandner, 2017), we conducted a sex and age-stratified analysis (18–64 years vs. ≥ 65 years). In addition, to reduce the risk of reverse causation, we conducted a sensitivity analysis stratifying those who reported being restricted by chronic disease in the past 6 months (‘yes’ vs. ’no’) and by BMI (kg/m^2^) (18.5–25.0; ‘normal weight’ vs. ≥ 25.0; ‘overweight/obese’).

## 3 Results

### 3.1 Sample description

The final sample was 23,635 (≥18 years). As shown in Table 1, 50.8% were female, 36.7% were aged 45–64 years and 20.2% had a ‘high’ socioeconomic status. Just under 70% rated their health as ‘*very good*’ or ‘*good*’, 44.3% had a ‘normal’ BMI (≥18.5–<25 kg/m^2^), 76.0% reported never smoking, and most (>90%) were classified as having non-hazardous alcohol consumption and not restricted by chronic disease in the past 6 months. Just under half reported meeting the aerobic guidelines and 67.0% reported having no significant depressive symptoms. A total 58.3% reported muscle-strengthening exercise 0 times/week, 12.3% 1 time/week, 12.1% 2 times/week, 10.3% 3–4 times/week, and 6.9% ≥5 times/week. A total of 40.4%, 39.9%, 11.0% and 8.6% reported ‘*good*’, ‘*fair*’, ‘*poor*’ and ‘*very poor*’ sleep quality, respectively.

**Table 1:** Sample characteristics, muscle-strengthening exercise and sleep quality^a^ of the German Health Update 2014.

		**n**
**Total**		23,635
**Sex**		**Weighted %**^b^**(n)**
	Male	49.2 (10,735)
	Female	50.8 (12,900)
**Age (years)**		
	18–29	17.1 (3,866)
	30–44	22.4 (5,298)
	45–64	36.7 (8,905)
	≥65	23.8 (5,566)
**Socioeconomic status**^c^		
	Low	19.9 (3,980)
	Medium	59.9 (13,245)
	High	20.2 (6,592)
**Self-rated health**		
	Very good	14.9 (3,774)
	Good	53.7 (12,824)
	Moderate	26.0 (5,797)
	Poor	4.7 (933)
	Very poor	0.7 (140)
**Body Mass Index (kg/m^2^)**		
	Underweight (<18.5)	1.8 (439)
	Normal (≥18.5–<25)	44.3 (10,871)
	Overweight (25–<30)	35.8 (8,156)
	Obese (≥30)	18.0 (3,957)
**Smoking status**		
	Never	76.0 (18,268)
	Occasional	5.4 (1,338)
	Daily	18.6 (3,980)
**Hazardous alcohol consumption**^d^		
	Yes	4.7 (1,111)
	No	95.3 (22,132)
**Restricted by chronic disease in the past 6 months**		
	Yes	6.5 (1,352)
	No	93.5 (22,034)
**Aerobic MVPA level (minutes/week)**		
	0–149 min/week ‘inactive’	54.7 (12,022)
	≥150 min/week ‘active’	45.3 (10,800)
**Depressive symptom severity**^e^		
	No significant depressive symptoms	67.0 (15,877)
	Mild depressive symptoms	22.9 (5,231)
	Moderate	6.8 (1,477)
	Moderately severe/severe	3.3 (674)
**Muscle-strengthening exercise (times/week)**		
	0	58.3 (13,337)
	1	12.3 (3,085)
	2	12.1 (2,990)
	3–4	10.3 (2,531)
	≥5	6.9 (1,662)
**Sleep Quality**^a^		
	Good	40.4 (9,617)
	Fair	39.9 (9,507)
	Poor	11.0 (2,521)
	Very poor	8.6 (1,894)

a Sleep Quality assessed by response to a single item question: “*Over the last 2 weeks, how often have you had trouble falling or staying asleep, or sleeping too much*”. Response options were: (i) ‘*Not at all*’ (good); (ii) ‘*On some days*’ (fair); (iii) ‘*More than half of the days*’ (poor); and (iv) ‘*Almost every day*’ (very poor).

b Sample weights provided by the GEDA 2014 (6).

c Socioeconomic status (low, medium and high) calculated by German-specific ‘SES Index’(12); and includes information on formal education/vocational training, occupational status and equivalenced to net household income

d Hazardous alcohol consumption classified as >20 g pure alcohol daily for women, and >40 g daily for men.

e Assessed by eight-item Patient Health Questionnaire depression scale (PHQ-8).

### 3.2 Prevalence ratios for sleep quality by weekly frequency of muscle-strengthening exercise

The unadjusted and adjusted prevalence ratios (APR) for decreasing levels of sleep quality by weekly frequency of muscle-strengthening exercise (reference = 0 times/week) are shown in Table 2. Associations were similar after adjusting for sociodemographic/lifestyle characteristics and aerobic MVPA. The APRs were lowest among those with ‘*very poor*’ sleep quality. For the total sample, in the fully adjusted model (‘Model C’), for ‘*fair*’ sleep quality, except for those reporting muscle-strengthening exercise ≥5 times/week (APR: 0.88; 95% CI: 0.80–0.96), all muscle-strengthening exercise frequencies were not significantly associated with lower APRs. Except for 2 times/week, all other muscle-strengthening exercise frequencies were significantly associated with a lower prevalence of ‘*poor*’ sleep quality (Range: 0.77–0.91). Among those reporting ‘*very poor*’ sleep quality, all muscle-strengthening exercise frequencies were significantly associated with a lower prevalence (Range: 0.57–0.70).

**Table 2:** Prevalence ratios (PR)^a^ of sleep quality^b^ according to weekly frequency of muscle-strengthening exercise (MSE) among German adults from the in the German Health Update 2014 survey (n = 23,602).

Sleep quality^b^	MSE (times/week)	Model A^c^ Prevalence ratio (95% CI)	Model B^d^ Prevalence ratio (95% CI)	Model C^e^ Prevalence ratio (95% CI)
Fair^f^	0	1 (reference)	1 (reference)	1 (reference)
	1	1.00 (0.94–1.06)	0.99 (0.93–1.05)	0.99 (0.93–1.06)
	2	1.03 (0.97–1.10)	1.03 (0.96–1.09)	1.03 (0.97–1.04)
	3–4	0.97 (0.91–0.95)	0.97 (0.90–1.09)	0.97 (0.90–1.10)
	≥5	0.88 (0.80–0.95)	0.87 (0.80–0.95)	0.88 (0.80–0.96)
Poor^f^	0	1 (reference)	1 (reference)	1 (reference)
	1	0.83 (0.73–0.94)	0.82 (0.72–0.93)	0.83 (0.73–0.94)
	2	0.90 (0.79–1.02)	0.90 (0.79–1.02)	0.91 (0.80–1.03)
	3–4	0.82 (0.72–0.94)	0.82 (0.71–0.94)	0.82 (0.71–0.94)
	≥5	0.77 (0.65–0.90)	0.78 (0.65–0.92)	0.77 (0.65–0.91)
Very Poor^f^	0	1 (reference)	1 (reference)	1 (reference)
	1	0.60 (0.51–0.70)	0.57 (0.48–0.67)	0.57 (0.47–0.67)
	2	0.69 (0.59–0.80)	0.69 (0.59–0.81)	0.70 (0.59–0.81)
	3–4	0.60 (0.59–0.80)	0.59 (0.49–0.71)	0.58 (0.48–0.70)
	≥5	0.69 (0.57–0.83)	0.71 (0.58–0.85)	0.68 (0.55–0.83)

a Prevalence ratio calculated using Poisson regression with a robust error variance.

b Sleep Quality assessed by response to a single item question: “*Over the last 2 weeks, how often have you had trouble falling or staying asleep, or sleeping too much*”. Response options were: (i) ‘*Not at all*’ (good); (ii) ‘*On some days*’ (fair); (iii) ‘*More than half of the days*’ (poor); and (iv) ‘*Almost every day*’ (very poor).

c Model A: unadjusted PR.

d Model B: adjusted PR: sex, age, socioeconomic status, hazardous alcohol consumption, self-rated health, body mass index, being restricted by chronic disease in the past 6 months and depressive symptom severity.

e Model C: adjusted for Model B + aerobic MVPA.

f Reference category = good sleep quality.

### 3.3 Sensitivity analyses

Supplementary Table 1 shows the APRs for reporting ‘*very poor*’ sleep quality stratified by sex, age, being restricted by chronic disease and BMI by weekly frequency of muscle-strengthening exercise. Overall, the APRs were generally similar for males vs. females, 18–64 vs. ≥ 65 years, and ‘normal BMI’ vs. ‘overweight/obese’ BMI. When stratified by being restricted by chronic disease, among those responding ‘yes’, none of the muscle-strengthening exercise were associated with a significantly lower APR.

## 4 Discussion

Compared to those doing none, any muscle-strengthening exercise was generally associated with a lower prevalence of ‘*poor*’ to ‘*very poor*’ sleep quality, with associations stronger across decreasing levels of sleep quality. All associations remained after adjustment for sociodemographic/lifestyle-related factors (e.g. sex, age, socioeconomic position, alcohol, smoking, BMI and chronic disease) and aerobic MVPA.

Currently, most epidemiological evidence on the benefits of physical activity on sleep quality is based on aerobic MVPA (U.S. Department of Health and Human Services, 2018). To our knowledge, this study provides the first population-level evidence favourably linking muscle-strengthening exercise to sleep quality. While the cross-sectional nature of the current study limits assumptions on cause-and-effect associations, our findings are supportive of a recent systematic review of brief/small-scale clinical exercise interventions that have identified compared to no exercise, muscle-strengthening exercise enhanced sleep quality (Kovacevic et al., 2018, Jurado-Fasoli et al., 2020). Within the context of the present study, we are unable to establish the mechanisms to explain these associations. However, a meta-analysis of clinical exercise studies have shown that muscle-strengthening exercise enhances cardiometabolic (e.g. improved glucose/lipid metabolism, insulin sensitivity, blood pressure) (Ashton et al., 2018), mental (e.g. reducing symptoms of anxiety/depression) (Gordon et al., 2018, Gordon et al., 2017) and functional health-related outcomes (e.g. enhanced mobility/muscle strength) (Liu, 2009), all of which are likely to be beneficial for sleep quality (Grandner, 2017).

A further key finding was that the APRs were lowest among those reporting the poorest quality sleep (‘*very poor*’). Additionally, among those reporting ‘*very poor*’ sleep quality, the APRs were similar among those doing muscle-strengthening exercise 1–2 times/week (APR: 0.57–0.70), compared to those doing 3–4 and ≥ 5 times/week (APR: 0.58–0.68). This lack of dose–response was surprising and does not follow the key exercise prescription principle of progressive overload (American College of Sports Medicine, 2009). However, given the cross-sectional nature of these data, we urge caution in drawing strong causal inferences. Nonetheless, this finding could indicate that small-to-moderate increases in muscle-strengthening exercise at the population-level may be an important lifestyle modification among those who routinely experience poor quality sleep. This is important considering ~60% of the sample reported no muscle-strengthening exercise, hence there is great scope to prompt greater proportions of the population engaged in this physical activity mode. Moreover, muscle-strengthening exercise offers an alternative form of physical activity/exercise among those with poor quality sleep, who might find it difficult to engage in and/or not enjoy aerobic MVPA.

The multiple sensitivity analyses suggested that the key findings of the present study remained consistent after stratification for sex, age and BMI. Interestingly, compared to those who reported being restricted by chronic disease in the past six months, the associations between muscle-strengthening exercise and sleep quality were more favourable among those who did not report being restricted by chronic disease. While these findings need to be confirmed in well-controlled longitudinal studies, this finding provides some indication that the associations between muscle-strengthening exercise and sleep quality observed in the current study might be causal.

A key strength was the recruitment of a large population sample of German adults. A further strength was the usage of standardised measures of muscle-strengthening exercise (Finger et al., 2015), sleep quality (Kroenke et al., 2009), and socioeconomic position (Lampert et al., 2013). The inclusion of an assessment of muscle-strengthening exercise is also a strength, which despite being in the global physical activity guidelines since 2010 (World Health Organization, 2010), is still seldom assessed in physical activity epidemiology.

As noted, the key limitation is that the cross-sectional design restricts interpretations of causality. A further limitation was the use of self-report assessments of sleep quality and muscle-strengthening exercise, which may have resulted in recall bias (e.g. social desirability, or over/under-reporting). Another important limitation was the modest GEDA 2014 response rate of 27.6%. Compared to non-responders, responders are more likely to be active and have an interest in their health and well-being. Therefore, until future studies with more representative samples are conducted, we urge caution in assuming that the key findings observed here are generalisable to the German population. This study is also limited by the crude assessment of sleep quality. The PHQ item requires the reporting of several sleep quality constructs grouped within a broad definition (‘sleeping too much’ ‘trouble falling asleep’ and ‘trouble staying asleep)’. Future studies may wish to examine these constructs separately to obtain a more nuanced association between sleep quality and muscle-strengthening exercise. Other limitations included the non-assessment of sleep medication usage, sedentary behaviour and the possibility that other unmeasured elements may have influenced results.

## 5 Conclusion

Among a population sample of German adults, muscle-strengthening exercise was associated with a lower likelihood of poor sleep quality. Although prospective studies are necessary to determine the direction of these associations, this preliminary epidemiological evidence suggests that successful public health campaigns that promote and support muscle-strengthening exercise may improve sleep quality at the population-level.

## Financial disclosure

All authors declare that there is no financial relationships with any organizations that might have an interest in the submitted work.

## Declaration of Competing Interest

The authors declare that they have no known competing financial interests or personal relationships that could have appeared to influence the work reported in this paper.
