# Supplementary material for: Muscle-strengthening exercise and sleep quality among a nationally representative sample of 23,635 German adults
Source: Prev Med Rep. 2020 Nov 25;20:101250. doi: 10.1016/j.pmedr.2020.101250 (PMC7718168; doi:10.1016/j.pmedr.2020.101250)
Supplement: Supplementary data 1 [file mmc1.docx]

| **Supplementary Table 1:** Adjusted prevalence ratios^a^ (APR) of sleep quality^b^ according to levels of weekly frequency of muscle-strengthening exercise (MSE): stratified by sex, restricted by chronic disease in the last 6 months and body mass index. | | | |
| --- | --- | --- | --- |
|  |  | **Sex** | |
| **Sleep quality ^b^** | **MSE (times/week)** | **Males APR^d^ (95% CI)** | **Females APR^d^ (95% CI)** |
| Fair^c^ | 0 | 1 (reference) | 1 (reference) |
|  | 1 | 0.95 (0.85-1.05) | 0.95 (0.80-1.05) |
|  | 2 | 1.04 (0.94-1.14) | 1.01 (0.93-1.10) |
|  | 3-4 | 0.96 (0.87-1.06) | 0.98 (0.89-1.08) |
|  | ≥ 5 | 0.92 (0.81-1.06) | 0.87 (0.76-0.99) |
| Poor^c^ | 0 | 1 (reference) | 1 (reference) |
|  | 1 | 0.73 (0.57-0.93) | 0.80 (0.68-0.93) |
|  | 2 | 0.80 (0.64-0.99) | 0.92 (0.79-1.08) |
|  | 3-4 | 0.78 (0.62-0.97) | 0.86 (0.72-1.03) |
|  | ≥ 5 | 0.83 (0.65-1.06) | 0.78 (0.61-0.99) |
| Very Poor^c^ | 0 | 1 (reference) | 1 (reference) |
|  | 1 | 0.50 (0.35-0.69) | 0.54 (0.44-0.66) |
|  | 2 | 0.50 (0.35-0.67) | 0.76 (0.63-0.92) |
|  | 3-4 | 0.60 (0.45-0.79) | 0.59 (0.46-0.74) |
|  | ≥ 5 | 0.59 (0.41-0.81) | 0.81 (0.62-1.03) |
|  |  | **Age** | |
|  |  | **18-64 years APR^e^ (95% CI)** | **≥65 years APR^e^ (95% CI)** |
| Fair^c^ | 0 | 1 (reference) | 1 (reference) |
|  | 1 | 0.99 (0.92-1.06) | 1.01 (0.87-1.17) |
|  | 2 | 1.02 (0.95-1.10) | 1.05 (0.91-1.22) |
|  | 3-4 | 0.97 (0.89-1.04) | 0.97 (0.83-1.13) |
|  | ≥ 5 | 0.89 (.079-0.99) | 0.87 (.074-1.01) |
| Poor^c^ | 0 | 1 (reference) | 1 (reference) |
|  | 1 | 0.83 (0.72-0.95) | 0.80 (0.58-1.08) |
|  | 2 | 0.93 (0.81-1.07) | 0.74 (0.52-1.02) |
|  | 3-4 | 0.82 (0.70-0.95) | 0.82 (0.59-1.11) |
|  | ≥ 5 | 0.82 (0.67-1.01) | 0.70 (0.50-0.94) |
| Very Poor^c^ | 0 | 1 (reference) | 1 (reference) |
|  | 1 | 0.74 (0.57-0.93) | 0.61 (0.41-0.87) |
|  | 2 | 0.61 (0.50-0.75) | 0.61 (0.41-0.88) |
|  | 3-4 | 0.72 (0.60-0.86) | 0.48 (0.30-0.72) |
|  | ≥ 5 | 0.56 (0.46-0.68) | 0.56 (0.38-0.78) |
|  |  | **Felt restricted by chronic disease in the last 6 months** | |
|  |  | **No APR^e^ (95% CI)** | **Yes APR^e^ (95% CI)** |
| Fair^c^ | 0 | 1 (reference) | 1 (reference) |
|  | 1 | 0.99 (0.93-1.06) | 1.19 (0.76-1.78) |
|  | 2 | 1.02 (0.96-1.09) | 1.16 (0.83-1.59) |
|  | 3-4 | 0.97 (0.90-1.04) | 1.06 (0.71-1.53) |
|  | ≥ 5 | 0.86 (0.79-0.95) | 1.10 (0.77-1.53) |
| Poor^c^ | 0 | 1 (reference) | 1 (reference) |
|  | 1 | 0.85 (0.74-0.97) | 1.05 (0.57-1.77) |
|  | 2 | 0.92 (0.81-1.05) | 0.87 (0.52-1.38) |
|  | 3-4 | 0.81 (0.70-0.94) | 1.10 (0.71-1.64) |
|  | ≥ 5 | 0.75 (0.62-0.89) | 0.99 (0.62-1.50) |
| Very Poor^c^ | 0 | 1 (reference) | 1 (reference) |
|  | 1 | 0.61 (0.51-0.73) | 1.00 (0.63-1.55) |
|  | 2 | 0.72 (0.60-0.86) | 1.02 (0.63-1.55) |
|  | 3-4 | 0.60 (0.49-0.73) | 0.93 (0.64-1.31) |
|  | ≥ 5 | 0.70 (0.55-0.87) | 0.76 (0.47-1.15) |
|  |  | **Body mass index (kg/m^2^)** | |
|  |  | **18.5-25.0 ‘normal weight’**  **APR^e^ (95% CI)** | **≥25.0 ‘overweight/obese’**  **APR^e^ (95% CI)** |
| Fair^c^ | 0 | 1 (reference) | 1 (reference) |
|  | 1 | 1.02 (0.93-1.11) | 0.98 (0.89-1.07) |
|  | 2 | 1.06 (0.97-1.15) | 1.01 (0.92-1.11) |
|  | 3-4 | 1.01 (0.92-1.10) | 0.94 (0.84-1.04) |
|  | ≥ 5 | 0.85 (0.75-0.97) | 0.90 (0.79-1.02) |
| Poor^c^ | 0 | 1 (reference) | 1 (reference) |
|  | 1 | 0.82 (0.68-0.99) | 0.80 (0.66-0.97) |
|  | 2 | 0.91 (0.75-1.08) | 0.89 (0.74-1.07) |
|  | 3-4 | 0.88 (0.72-1.06) | 0.75 (0.60-0.92) |
|  | ≥ 5 | 0.76 (0.59-0.96) | 0.81 (0.63-1.03) |
| Very Poor^c^ | 0 | 1 (reference) | 1 (reference) |
|  | 1 | 0.60 (0.46-0.76) | 0.70 (0.52-0.92) |
|  | 2 | 0.69 (0.53-0.87) | 0.64 (0.50-0.82) |
|  | 3-4 | 0.57 (0.43-0.74) | 0.76 (0.61-0.94) |
|  | ≥ 5 | 0.70 (0.52-0.93) | 0.70 (0.52-0.92) |
| ^a^ Prevalence ratio calculated using Poisson regression with a robust error variance.  ^b^ Sleep Quality assessed by response to a single item question: “*Over the last 2 weeks, how often have you had trouble falling or staying asleep, or sleeping too much*”. Response options were: (i) ‘Not at all’ (Good); (ii) ‘On some days’ (Fair); (iii) ‘More than half of the days’ (Poor); and (iv) ‘Almost every day’ (Very Poor).  ^c^ Reference category = good sleep quality.  ^d^ Age, socioeconomic status, hazardous alcohol consumption, self-rated health, body mass index, being restricted by chronic disease in the past 6 months and depressive symptom severity.  ^e^ Sex, socioeconomic status, hazardous alcohol consumption, self-rated health, body mass index, being restricted by chronic disease in the past 6 months and depressive symptom severity.  ^f^ Age, sex, socioeconomic status, hazardous alcohol consumption, self-rated health, body mass indexb and depressive symptom severity.  ^g^ Age, sex, socioeconomic status, hazardous alcohol consumption, self-rated health, being restricted by chronic disease in the past 6 months and depressive symptom severity. | | | |
